# The COVID-19 Pandemic: Healthcare Crisis Leadership as Ethics Communication

**DOI:** 10.1017/S0963180120000444

**Published:** 2020-05-22

**Authors:** MATTI HÄYRY

**Keywords:** COVID-19, pandemic, bioethics, liberal democracy, social democracy, Finland, Sweden, utilitarianism, republicanism, lying for benevolent reasons

## Abstract

Governmental reactions to crises like the COVID-19 pandemic can be seen as ethics communication. Governments can contain the disease and thereby mitigate the detrimental public health impact; allow the virus to spread to reach herd immunity; test, track, isolate, and treat; and suppress the disease regionally. An observation of Sweden and Finland showed a difference in feasible ways to communicate the chosen policy to the citizenry. Sweden assumed the herd immunity strategy and backed it up with health utilitarian arguments. This was easy to communicate to the Swedish people, who appreciated the voluntary restrictions approach and trusted their decision makers. Finland chose the contain and mitigate strategy and was towards the end of the observation period apparently hesitating between suppression and the test, track, isolate, and treat approach. Both are difficult to communicate to the general public accurately, truthfully, and acceptably. Apart from health utilitarian argumentation, something like the republican political philosophy or selective truth telling are needed. The application of republicanism to the issue, however, is problematic, and hiding the truth seems to go against the basic tenets of liberal democracy.

The COVID-19 pandemic has posed three questions concerning our common goals and the best ways of reaching them. What kind of a society do we want to live in? What kind of a political system do we want to endorse? What can governments do to secure us what we want in times of crisis? As I have observed, in the commercial and social media, people’s reactions to the pandemic and governmental attempts to control it,^1^ the societal values that keep cropping up are openness, equality, autonomy, and solidarity. People in liberal democracies believe that these values are best fostered by their own system and its main elements: rule of law, respect for basic rights, transparent political decision making, and participatory governance. Social democracies abide by the same rules but put more emphasis on the common good and may assume a more lenient stand on restrictions of individual freedom.

In situations like the COVID-19 pandemic, governments have what appear to be conflicting duties. They are responsible for the safety and basic wellbeing of their people, but what promotes health may be detrimental to businesses and livelihoods; and vice versa. Governments are also responsible for safeguarding their citizens against major limitations of civic liberty and personal autonomy, but such limitations may be deemed necessary to slow down the spread of contagions. And they are responsible for treating all people equally, yet some instruments of pandemic control hit already vulnerable groups harder than the rest of the population. The elderly may be isolated in their own homes or care homes, ostensibly for their own good, but with potentially harmful consequences. The condition of patients waiting for nonemergency procedures may deteriorate, as hospitals are turned into intensive- and emergency-care units. People with disabilities and mental health issues may suffer.

We do not know what the right decisions during the pandemic are. We do know, however, that it is good if our governments have a decisive approach, and an approach that guides us through the current crisis as unscathed as possible. It is also important that we know what the chosen way is, and how our governments justify them ethically. This is why I think that the best crisis leadership could be ethics communication.

## The Four Main Approaches

The COVID-19 pandemic will be eventually conquered if researchers can develop an effective and safe vaccine and medical scientists can considerably improve the treatments. The vaccine would halt the spread of SARS-CoV-2, the virus that causes the disease. Perfect treatments would make the disease innocuous, at least in regions where they can be made available. We do not know if or when we can expect these to be achieved.^2^ In the meantime, four main approaches to dealing with the pandemic have offered themselves: (1) Containing and mitigating the disease, (2) Letting the disease spread until herd immunity is reached, (3) Testing, tracking, isolating, and treating, and (4) Suppressing the disease. Methods (1), (3), and (4) are closely related and can be used consecutively or in combination. All strategies have their risks and benefits, although knowledge of these is not accumulating straightforwardly due to the complex variables and regional differences involved.^3^

### Contain and Mitigate

Since the virus transmits between people primarily in close physical contact, *containing* the disease and its spread involves strict regulation on people’s movement, gatherings, and involvement with one another. The methods include restricting movement between regions; closing down national borders; setting curfews; implementing lockdown, isolation, and quarantine rules; moving to distant working; closing daycare centers, schools, and universities; cancelling mass events; and shutting down bars, restaurants, and nonessential services. In most countries that reacted to the pandemic in an orderly fashion, one combination or another of these was initially endorsed. The primary aim of containment is to *mitigate* the effect of the pandemic by “flattening the curve,” that is, by not letting too many people have the disease at the same time. This gives healthcare systems time to prepare and a better chance to provide effective care.

The legality and morality of many of these methods have been questioned.^4^ Are governments in liberal democracies entitled to apply such intrusions on the lives of their citizens? Instructions and guidance are more readily acceptable, as they do not necessarily involve coercion or curtailments of liberty or autonomy. But what about physical restrictions backed up by the use of police or military force? Standard liberal views at least have shunned them, although they, too, can have their justifications.^5^ In between, citizens have in some cases interpreted public instructions as legal orders. After a few weeks into the containment stage, many people over 70 in Finland began to ask, “When are we allowed to see our grandchildren again?” The moral coercion of the public opinion had, apparently, worked, as the home isolation of the elderly had never been more than a governmental recommendation.^6^

### Build Up Herd Immunity

The second alternative is to let the disease spread until *herd immunity* is reached. When a sufficient percentage (a vast majority) of the population has been infected, the virus cannot find new hosts, and it will peter out, at least until a new mutation starts to circulate. The leaders of the United Kingdom and the United States have flirted with this idea, but their overall strategies have remained unclear. Sweden, however, seems to have embraced the approach, with considerably fewer restrictions and controls than its neighboring countries. This response may have been partly dictated by a late awakening to the situation, but the government and other public officials have stood by it since.^7^ Sweden’s state epidemiologist Anders Tegnell has argued that the death rate, appalling in Sweden during the first few months,^8^ will be matched by other countries later, when the second wave of the pandemic hits the populations that are first protected and then gradually left unprotected again.^9^

The herd immunity approach raises both scientific and moral questions.^10^ Since SARS-CoV-2 is a new virus, we cannot be sure that those infected develop a proper and lasting immunity, especially if the virus begins to mutate significantly. Since tests have been unreliable, we cannot know for certain that our figures are correct and that herd immunity can be reached within a few months, as the Swedish state epidemiologist believes. Also, as the number of COVID-19 deaths in Sweden is really remarkably high, effective vaccinations and better treatments may yet save countries that have chosen containment from such figures. An additional moral concern is solidarity, which seems to have taken a blow with the disproportionate number of deaths among the elderly.

### Test, Track, Isolate, and Treat

The countries that initially responded by containment and mitigation eventually have to make a further choice. When the basic reproduction number *R*_0_ has been forced down to less than 1 by handwashing, facemasks, physical distancing, and restrictions, one infected person infects less than one other, and the pandemic begins to fade. If all precautions and restrictions are abandoned at once, however, *R*_0_ again rises above one, and the pandemic starts to respread. One strategy in the face of this, adopted by Germany and Finland among others, is to *test*, *track*, *isolate*, and *treat.* The number of tests is increased considerably, and governments and technology companies are developing smart phone applications that make it possible to follow infection routes, identify people who have been in contact with carriers, test them, and, depending on the test result, isolate or treat them. The spread of the pandemic is allowed in this model for public health or other reasons, but under strict scrutiny.

This line of action is justified by the public health consideration that it is better to have the infections spread moderately and in a controlled manner than to stop the pandemic for now and experience an equally lethal second wave in a few months’ time. The strategy is clouded, however, by several concerns. The tests appear to be far from reliable, and the surveillance of the population by yet another data-collecting device raises suspicions.^11^ Apart from these, we have to ask what the approach is aiming to achieve. If the *R*_0_ number is kept close to 1, which would be safer than letting it rocket, it could take years before the pandemic is over. A safe and effective vaccine could provide the solution, but we do not know if or when to expect its completion. Meanwhile, economies can suffer a great deal. Although scenarios and projections^12^ of the potential economic damage differ and can be based on partisan interests, ideological premises, and imperfect information, the recession caused by the pandemic is a fact that cannot be ignored.

### Suppress

The containment and mitigation approach, supplemented by testing, tracking, isolating, and treating, has a glitch that may or may not be predominantly semantic. This is where we get to the importance of ethics communication in crisis leadership.

In its nonsemantic form, the issue is that by deliberately letting the pandemic spread governments seem to assume the herd immunity strategy. If this is implemented quickly and effectively, the Swedish state epidemiologist’s glum prediction could become accurate. Countries choosing this route would end up having a death toll similar to Sweden’s.

In response to this, some scientists have suggested that the disease should be *suppressed* rather than contained. The aim would be a near-complete eradication of the disease from certain regions.^13^ One Finnish academic lawyer used the example of tuberculosis, which appears in Finland from time to time but does not at the moment present a major threat to public health in the country.^14^

The choice of example shows a weakness in this kind of thinking. Although the primary duty of any government is to its own citizenry, pandemics are a global phenomenon. Tuberculosis is still a major health issue in many third-world countries, and it has a slightly callous ring to it if we say that the eradication of a disease from the first world provides an adequate cure to the problem.

Be that as it may, other questions emerge. We may have to wait for the vaccine for 2 or 3 years. Are we prepared to live in lockdown societies, with the economic implications, for so long? What if scientists do not find the vaccine? What if the virus mutates?

The other issue is that the semantics here are unclear. Neil Ferguson and coworkers at the Imperial College London suggested earlier on that suppression is the only viable strategy in the United Kingdom and in the United States.^15^ But they then went on to say that suppression can be turned on and off as the situation evolves, so that if intensive-care units are overcrowded, restrictions can be reinstated. This sounds like the confine, mitigate, test, track, isolate, and treat policy that Germany and Finland have assumed. Whatever name we give to the approach, however, the way forward outlined by Ferguson and his team seems to lead back, if medicine is the measure, to building up herd immunities, not to holding the *R*_0_ < 1 lockup steadfastly until a vaccine is developed.

## Ethics Communication

The question of naming the implemented policy accurately and without delay goes far beyond semantics. A seemingly innocuous example from Finland elucidates this. In the beginning of May 2020, the curve had been successfully flattened, the basic reproductive value *R*_0_ was below 1, and the government considered lifting some restrictions and revising the general guidelines for physical distancing.^16^ The government made its decisions and aired them live on television and via other media. A couple of days later, the Finnish Ministry of Social Affairs and Health publicized an announcement, listed government’s decisions, and described the new approach as a test, track, isolate, and treat model to *manage* and *control* the pandemic. Prime Minister Sanna Marin was not satisfied by the wording and corrected that the government still aims to *suppress* the pandemic. The Prime Minister’s correction was supported by the law, as it is the government’s legal duty to suppress pandemics, not to manage or control them.^17^ We do not know, however, what exactly motivated her proclamation. The Ministry bowed to the edict and revised their wording.^18^

The fact remains that the Ministry’s original formulation was probably closer to the truth. The government may want to talk about suppression for rhetoric reasons, but since this is only “suppression” in the Imperial College sense, the actual strategy seems to be confinement to mitigate damage to public health and possibly to national economy. It is definitely not what Iceland, an isolated island with a population of 364 000, has done by forcing the number of new contagions to zero.^19^

### The Health Utilitarian Case—Difficult to Communicate When the Worst Is Over

During the first stage of pandemic control, governments with a relatively clear plan like Sweden and Finland had an easy job communicating their actions to the citizenry. Both herd immunity and containment policies were, early on, effortlessly verbalized and rationalized in health utilitarian terms. The Public Health Agency of Sweden and the Finnish Institute for Health and Welfare produced recommendations based mainly on epidemiological calculations, politicians made decisions according to the recommendations, and key ministers announced them to the general public as inevitable medical truths. Sweden can continue on this path, as the lighter restrictions policy enables more business activities and silences the loudest opposition from economic circles. Finland is a different matter, as are all other countries that are considering postcontainment ways of living through the pandemic.

When a choice has to be made between continued containment and fully-blown suppression, epidemiology does not give unequivocal answers anymore. We simply do not know which choice will, in the end, be the best life saver, health promoter, or quality adjusted life year (QALY) producer overall. Nor do we know how to weigh and balance lives, health, and QALYs. Other values further complicate the matter. If the economy does not work, citizens will experience the adverse effects in their lives, possibly for years. And there are environmental, ecological, cultural, and political values which may be threatened by public policy choices. Since most of these are not commensurable, utilitarian or other straightforwardly consequentialist decisions cannot be made, let alone communicated accurately to the general public.

The most severe obstacle for utilitarian truth telling, beyond the period of clear and immediate danger to all, is that the message is bound to be unpalatable to parts of the population. If the government goes for continued confinement to revitalize the economy, telling the truth would also require them to predict how many lives would be lost as a consequence. People would then argue that the sacrifice is immoral and point out that the lives lost would be in vulnerable groups. If the government goes, instead, for genuine suppression, telling the truth would require them to estimate the remaining time for the restrictions, their effects on the economy, and their impact on people’s wellbeing. Businesspeople would object, others would complain about lost livelihoods, and the rest would begin to protest against the curtailment of their liberty.

Note that I do not present a criticism against health utilitarianism here. I and others have done that decades ago and repeated it more recently.^20^ ^,^^21^ ^,^^22^ ^,^^23^ ^,^^24^ The point here is that insofar as healthcare crisis leadership is ethics communication, health utilitarianism cannot be presented to the public accurately, truthfully, and acceptably and all at once. Governments moving along from the initial pandemic control to ongoing confinement and mitigation or full suppression of the virus from their region have to find a better truthful narrative or consider not telling the truth, or at least the whole truth, to begin with.

### Presenting Truthfully the Republican Case

Republican political philosophy could provide a better story.^25^ We can use as a starting point the observation that governments do not live lives or conduct businesses. People in the civil society do these things, and the role of governments is to see to it that the common good is best served by people living their lives and businesspersons conducting their businesses. This involves, especially in exceptional situations like the COVID-19 pandemic, restrictions of civil liberties. Insofar as we believe that liberties are intrinsically valuable and something to be protected, we can only curtail them if we have a proper justification.

Those who define liberty and freedom as noninterference on choices and actions can point out that the pandemic policies of confinement and suppression interfere with lives and businesses in numerous ways. And this is not restricted to pandemics. As long as some state of emergency is on, liberal and authoritarian regimes do not seem to differ all that much. Some have gone on to argue that modern societies are characterized by a constant state of emergency. This would mean that the difference between democracy and totalitarianism is tenuous and the value of our cherished liberal democracy questionable.

The republican, at this point, comes to the rescue, suggesting that we should define liberty differently, as nondomination. We are free in the sense of nondomination when we are ourselves, as citizens, in control of the power imposing the interferences. No external tyrants then subject us to their arbitrary rule. The power emanating from us is channeled through our democratic leaders to design only the most adequate restrictions to achieve the common good that we all understand and accept as our joint goal. Trust in our government then translates into our obeying all the pandemic-related as well as other public orders and instructions as expressions of our own will,^26^ while we can still embrace freedom and reject totalitarian forms of government.

Since being stopped on a border by a republican police officer does not necessarily feel very different from being stopped by an authoritarian one, however, we need further assurances. The standardly recognized ones are guaranteed temporariness, transparent information, and everybody’s benefit. Temporariness requires that emergencies are an exception and not a rule. Even amidst a pandemic, authorities should be constantly reviewing the situation and removing bans and restrictions where they are no longer absolutely needed. Most governments, democratic and authoritarian alike, at least give the impression of doing this with their actions during the COVID-19 crisis. The transparency of the information conveyed to the media and the general public is an issue in which a dividing line may exist. China and Russia, not to mention North Korea, can be trusted to give out scant information in a way that does not embarrass their leaders. At the other end of the continuum, Belgium, Denmark, Finland, France, Iceland, Italy, Germany, Norway, and Sweden have communicated in a more transparent manner, although the clarity of the message is not always certain. The unclarity is mostly due to inadequacies in the information that the authorities have. So it seems that liberal democracy does have an edge over totalitarian regimes in the light of the first two specifications to the idea of freedom as nondomination.

How about the third caveat, then, that everybody should benefit? As things in all modern societies are, this brings us back to the objection that already sank the health utilitarian boat. Currently, the extremely well-to-do benefit from almost all policies, the well-to-do benefit from most policies, the not-so-well-to-do benefit from some policies, and the not-at-all-well-to-do benefit from very few policies. As long as this is the general situation, disagreement remains a possibility. If governments announce truthfully plans to subsidize corporations so that their affluent stockholders are protected from losses, other factions of society may have legitimate grievances.

### Sweden or Lying for Benevolent Reasons?

The health utilitarian and republican narratives are not the only ones, but they are the ones most readily available to the leaders of liberal and social democracies. Alternatives include the war rhetoric, and it has been used widely, most audibly in the United Kingdom and in the United States. In naming a common enemy and giving purpose to the sacrifice of the heroes who die at the claws of the enemy, it can keep up morale, but the lack of actual content may weaken its reception over time. Some countries and regions have also assumed the strategy of mitigating the problem, stating confidently that COVID-19 is just another flu, and noting that people die every year of influenza without the lockdown of societies. In the light of our current information, this is not an accurate or helpful comparison. But if these approaches are rejected, we seem to be led to a twofold conclusion.

First, it seems that the only country in the world in which the government can tell the truth as best they can and still command the respect of the vast majority of their citizens is Sweden. As an old no-nonsense eugenic social democracy,^27^ it can assume a health utilitarian line; and as a consensus-decision-making society, it appreciates that the authorities have not curtailed civil liberties without asking the people. Opposition exists, but the government and the epidemiologists have the upper hand for the time being. Swedes are, as a commentator put it, “trusting and unflappable.”^28^

Since other governments cannot perfectly replicate Sweden’s approach, they are left with the choice of doing what they see best and being selective with the truth in their communications with the citizenry.^29^ Lying for benevolent reasons, shunned as it is, may be the only realistic way forward, when countries like Germany begin to remove the restrictions purposefully and methodically.^30^ Apart from the potentially dubious moral philosophy, though, this is not necessarily a route that can be travelled more than once.^31^ The Finnish example of presenting recommendations to the elderly in a form that could be interpreted as legal orders and prohibitions is a case in point. Now that the over 70s know, after media exposure, that the government may have overstated their case, people are prone to be more cautious.

This brings us back to the premise of my narrative. If we want to live in liberal democracies, and if liberal democracies gain our trust by nurturing principles like openness and transparency, is mispresenting the truth, or hiding the fact that we do not know the truth, ever the right answer? If it is not, then I am left with this disconcerting question: Do citizens need better liberal democracy or do liberal democracies need better citizens?

## Notes

1. During March 26–April 26, 2020, I followed, through my customary media and social media feeds, the development of the COVID-19 pandemic, the actions that the government of Finland took to contain it, and the public reactions of Finnish researchers whose work has to do with bioethics. I chronicled the results, and they were published on 30 April—see note 4, Häyry 2020. After this, I continued to follow the situation in Finland (which was moving forward from the first containment period) and Sweden (Finland’s neighboring country with a different, less restrictive approach to handling the pandemic). I also started to follow the international bioethics discussion on the topic. I paid special attention to the governments’ communication of the ethical bases for their decisions. Ethics communication began to emerge as the leadership solution in times of crisis, and I incorporated the idea into my observation. The data, as far as my topic and angle were concerned, saturated in a week, during May 7–14, 2020, after which I compiled this report. The references are indicative more than exhaustive.

2. Branswell H. Mounting promises on Covid-19 vaccines are fueling false expectations, experts say. *STAT* 2020 May 6; available at https://www.statnews.com/2020/05/06/mounting-promises-on-covid-vaccines/?utm_content=bufferabe3f&utm_medium=social&utm_source=twitter&utm_campaign=twitter_organic&fbclid=IwAR1vR9K1o2oemAqWSuSB0bViH6_vaglwPn_eP8XO-qKHxbjRi5vnW_D6xe4 (last accessed 14 May 2020).

3. COVID-19 Pandemic; available at https://en.wikipedia.org/wiki/COVID-19_pandemic#Management (last accessed 14 May 2020).

4. Häyry M. The COVID-19 pandemic: A month of bioethics in Finland. *Cambridge Quarterly of Healthcare Ethics* 2020. doi:10.1017/S0963180120000432.

5.

Häyry M , Takala T. Coercion . ten Have H , ed. Encyclopedia of Global Bioethics. Cham: Springer; 2016 , at 595–605; available at https://it.b-ok2.org/book/2802341/8ecdbd (last accessed 14 May 2020).

6. Vuorelma J, Rydenfelt H. Uusi normaali: vakiintuuko moukarivalta? [The new normal: Will sledgehammer rule remain? in Finnish]. *Etiikka.fi* 2020 May 8; available at https://etiikka.fi/uusi-normaali-vakiintuuko-moukarivalta/ (last accessed 14 May 2020).

7. Paterlini M. “Closing borders is ridiculous”: The epidemiologist behind Sweden’s controversial coronavirus strategy Anders Tegnell talks to Nature about the nation’s “trust-based” approach to tackling the pandemic. *Nature News Q&A* 2020 Apr 21; available at https://www.nature.com/articles/d41586-020-01098-x (last accessed 14 May 2020).

8. Kim S. Sweden’s coronavirus death toll “horrifying” says scientist behind country’s anti-lockdown strategy. *Newsweek* 2020 May 7; available at https://www.newsweek.com/sweden-coronavirus-deaths-children-lockdown-1502548?fbclid=IwAR2NO4yFH3cgfEI9pptaVbGwY8j3K_9L6yJQr6oY1uCh1DBWommJPSGO2Fk (last accessed 14 May 2020).

9. Milne R. Architect of Sweden’s no-lockdown strategy insists it will pay off. *Financial Times* 2020 May 8; available at https://www.ft.com/content/a2b4c18c-a5e8-4edc-8047-ade4a82a548d (last accessed 14 May 2020).

10. Dowdy D, D’Souza G. Early herd immunity against COVID-19: A dangerous misconception. *Johns Hopkins Coronavirus Research Center* 2020; available at https://coronavirus.jhu.edu/from-our-experts/early-herd-immunity-against-covid-19-a-dangerous-misconception?fbclid=IwAR3nEtbDHdXVPQy1-08rSUTDnfvdaiLX7luFRhTenvGtw90sP9HHy6lt-2I (last accessed 14 May 2020).

11. Turunen J. Valvooko meitä pian korona-isoveli? Kiistellystä valvonnan teknologiasta halutaan ratkaisu taudin hallintaan Suomessakin [Will the corona big brother control us soon? The contested surveillance technology is suggested as a solution to controlling the disease in Finland, in Finnish]. *Helsingin Sanomat* 2020 May 13; available at https://www.hs.fi/talous/art-2000006505692.html?fbclid=IwAR391ZXJ7Qu6LS1smUcd2GL5GfVxeG56994Mx5e34sfAen65Gy_R59zZmaA (last accessed 14 May 2020).

12. Anonymous. Report: Finland needs stimulus then austerity to get through coronavirus crisis. *YLE News* 2020 May 8; available at https://yle.fi/uutiset/osasto/news/report_finland_needs_stimulus_then_austerity_to_get_through_coronavirus_crisis/11343155 (last accessed 14 May 2020).

13. Anonymous. Tutkijaryhmä vetoaa Marinin hallitukseen: “Koronaviruksen tukahduttaminen on mahdollista ja se pitää tehdä nyt” [A group of researchers appeals to Marin’s government: “The suppression of the coronavirus is possible and should be done now, in Finnish]. *Uusi Suomi* 2020 May 7; available at https://www.uusisuomi.fi/uutiset/tutkijaryhma-vetoaa-marinin-hallitukseen-koronaviruksen-tukahduttaminen-on-mahdollista-ja-se-pitaa-tehda-nyt-lue-koko-avoin-kirje/586295ac-23b3-461f-81d9-bf835c45095d (last accessed 14 May 2020).

14. Kauppinen L. Mihin tietoon perustuu arvio siitä, ettei epidemiaa voi tukahduttaa pysyvästi? THL:n Mika Salminen ja bakteeriopin professori ovat eri linjoilla [On what information is the estimate that the epidemic cannot be permanently suppressed based? THL’s Mika Salminen and a professor of bacteriology disagree, in Finnish]. *Helsingin Sanomat* 2020 May 6; available at https://www.hs.fi/politiikka/art-2000006499119.html?fbclid=IwAR3MVzUc_laQUNy7jERGkib3Sn5jaF9vugENCz3vmJMY9yJBmbyMkeW9Z38 (last accessed 14 May 2020).

15. Anonymous. COVID-19 suppression “only viable strategy at the current time”. *MedicalNewsToday* 2020 Mar 27; available at https://www.medicalnewstoday.com/articles/covid-19-suppression-only-viable-strategy-at-the-current-time#Turning-suppression-off-and-on (last accessed 14 May 2020).

16. Teivainen A. Coronavirus has a reproduction rate of 0.8 in Finland, tells Marin. *Helsinki Times* 2020 May 5; available at https://www.helsinkitimes.fi/finland/finland-news/domestic/17626-coronavirus-has-a-reproduction-rate-of-0-8-in-finland-tells-marin.html (last accessed 14 May 2020).

17.
*Tartuntatautilaki* [*The law concerning contagious diseases*, in Finnish] 1227/2016; available at https://www.finlex.fi/fi/laki/alkup/2016/20161227?fbclid=IwAR13GwNSyd1J6ZNhTuAZ84qCqQddJ9LrJcEfEMxRArvQ1LDV0rx3uzLFedM (last accessed 14 May 2020).

18. Luukka T. Koronalinjauksen muoto muuttui perjantai-iltana [The wording for the corona approach was revised on Friday night, in Finnish]. *Helsingin Sanomat* 2020 May 10:A 16.

19. Pelli P. Islanti painoi uudet tartunnat nollaan [Iceland forced new contagions to zero, in Finnish]. *Helsingin Sanomat* 2020 May 14:A16.

20.

Harris J. QALYfying the value of life. Journal of Medical Ethics 1987;13 10.1136/jme.13.3.117PMC13756583669036:117–23.

21.

Häyry M , Häyry H. Health care as a right, fairness and medical resources. Bioethics 1990;4:1–21 1165087710.1111/j.1467-8519.1990.tb00062.x.

22.

Häyry M. Measuring the quality of life: Why, how and what? Theoretical Medicine 1991;12:97–116 189695610.1007/BF00489791.

23. Häyry M. Priorisointiin ei ole hyvä joutua mutta pitäisikö siitä puhua? [Prioritization would be bad, but should we talk about it? in Finnish]. *Etiikka.fi* 2020 Apr 8; available at https://etiikka.fi/priorisointiin-ei-ole-hyva-joutua-mutta-pitaisiko-siita-puhua/ (last accessed 14 May 2020).

24. Harris J. Why kill the cabin boy? *Cambridge Quarterly of Healthcare Ethics* 2020. doi:10.1017/S0963180120000420.

25. Laborde C. Liberty in the time of Corona. *OXPOL* 2020 May 7; available at https://blog.politics.ox.ac.uk/liberty-in-the-time-of-corona/?fbclid=IwAR0I-yesBoIH3EQhLpy7zCuJlbhrSDrAM1tuq3a0tVFyfGQc6sDdAETmU7M (last accessed 14 May 2020).

26. Cf. the “communitarian” and “conservative” views on coercion—see note 5, Häyry, Takala 2016, at 601–2.

27. Häyry M. The historical idea of a better race. *Studies in Ethics, Law, and Technology* 2008;2:1–26; available at https://www.researchgate.net/publication/40823116_The_Historical_Idea_of_a_Better_Race (last accessed 14 May 2020).

28. Gustavsson G. Has Sweden's coronavirus strategy played into the hands of nationalists? *The Guardian* 2020 May 1; available at https://www.theguardian.com/world/commentisfree/2020/may/01/sweden-coronavirus-strategy-nationalists-britain (last accessed 14 May 2020).

29. Juholin E, Rydenfelt H. Hyvä viestintä, parempi viestintäkulttuuri [Good communication, better communication culture, in Finnish]. *ProComma Academic* 2016:12–27; available at https://viestijat.fi/hyva-viestinta-parempi-viestintakulttuuri/#296df46c (last accessed 14 May 2020). Elisa Juholin and Henrik Rydenfelt argue that transparency or openness and potential harm to audiences or stakeholders weigh towards opposing conclusions in the ethical assessment of communication.

30. Mahlamäki H. Tartuttavuusluku nousi, mutta Saksa ei jarruta [The reproduction number went up, but Germany does not pull the brakes, in Finnish]. *Helsingin Sanomat* 2020 May 14:A 17.

31. Takala T, Häyry M. Benefiting from past wrongdoing, human embryonic stem cell lines, and the fragility of the German legal position. *Bioethics* 2007;21:150–9. In this article, we maintain that in changing their stem cell policy into the more liberal position, German legislators used arguments that can only be used once.

